# Computations of Confidence Intervals for Estimates in the United States National Hospital Discharge Survey, 1979–2000

**Published:** 2005-06-15

**Authors:** Yao-Hua Luo Luo, Matthew Zack

**Affiliations:** Division of Adult and Community Health, National Center for Chronic Disease Prevention and Health Promotion, Centers for Disease Control and Prevention; Division of Adult and Community Health, National Center for Chronic Disease Prevention and Health Promotion, Centers for Disease Control and Prevention, Atlanta, Ga

## Abstract

**Introduction:**

The National Hospital Discharge Survey is a primary data source for epidemiology research in the United States. To ensure that estimates are reliable, confidence intervals need to be calculated. The original survey data source is not available to the public, and the usual statistical methods are unsuitable for calculating confidence intervals. Instead, calculating confidence intervals requires using the statistical methods and relative standard errors that the U.S. National Center for Health Statistics has provided. However, the relative standard error parameters differ by hospital, patient category, and group. They also change yearly with sampling and are expressed differently before and during or after 1988. Consequently, manual computations of confidence intervals with multiple groups, diseases, and years are inefficient and prone to error. We developed a SAS program to compute confidence intervals for National Hospital Discharge Survey data from 1979 through 2000, newborns excluded.

**Methods:**

We transposed 22 tables of relative standard error parameters (one for each year) into two new parameter tables that maintain the sampling designs before 1988 and during and after 1988 but are similar in overall structure. We unified all values to make each set of relative standard error parameters unique. We developed a program, COMPURSE, to search for relative standard error parameters for inputted estimates and to calculate confidence intervals. We set up an interface program for users to enter data, time period, confidence interval level, and output location; to read the relative standard error parameter tables; and to run the COMPURSE program.

**Results:**

For different sets of National Hospital Discharge Survey data, COMPURSE efficiently and correctly retrieved relevant relative standard error parameters for estimates and accurately calculated relative standard errors, standard errors, and confidence intervals for annual estimates, multiple-year summaries, and average annual estimates.

**Conclusion:**

The program COMPURSE helps users analyze National Hospital Discharge Survey data efficiently.

## Introduction

The National Hospital Discharge Survey (NHDS) is a national probability survey designed to provide information on characteristics of inpatients discharged from nonfederal short-stay hospitals in the United States (1). The NHDS is a major data source for many health studies (2,3). Conducted annually by the U.S. National Center for Health Statistics (NCHS) since 1965, the NHDS collects data from about 270,000 inpatient records in a national sample of about 500 hospitals, which comprises about 1% of inpatients nationwide (1,4). The NHDS includes only hospitals with an average length of stay of fewer than 30 days for all patients, general hospitals, or children's general hospitals; the NHDS excludes federal, military, and Department of Veterans Affairs hospitals, hospital units of institutions (such as prison hospitals), and hospitals with fewer than six beds staffed for patient use (1). 

Because of complexities in survey sampling, calculating confidence intervals (CIs) for estimates extracted from the NHDS is necessary to determine whether these estimates are reliable. The original survey data source is not available to the public, and usual statistical methods are unsuitable for this calculation. Instead, calculating confidence intervals requires using the statistical methods and relative standard errors (RSEs) that the NCHS provides. RSE measures variability in estimates and is defined as the ratio of the standard error (SE) of the estimate to the estimate itself. In 2002, the NCHS issued a CD-ROM containing all data and documentation from the 1979–2000 NHDS (4). Such information provides a convenient source for summarizing data over multiple years. This CD-ROM has four parts: 1) hospital discharge data on newborns (NEWBORN) and other than newborns (NOTNB); 2) the corresponding annual civilian population data (Excel files) from the U.S. census summarized by race, sex, and geographic region; 3) annual tables (Excel files) from which the data user can calculate CIs; and 4) documentation for these data, including instructions on how to compute these SEs and CIs from the RSE.

Even with these instructions, however, it is difficult to search the current CD-ROM for proper parameters and to calculate CIs. In 1988, the NCHS changed its methods for estimating the RSEs, making these estimates more accurate and making it simpler to calculate CIs than before 1988 (4). However, these changes cause new difficulties in summarizing estimates from 1979 through 1987 with those occurring afterward because the annual RSE parameter tables on the CD-ROM differ for these two periods in four ways (Table 1). The first difference is that the types of statistics for hospitalizations differ in their number and form. Before 1988, three types of statistic are listed as subtables: the first-listed diagnosis or all-listed diagnoses, days of care, and procedure. Starting in 1988, four types of statistic are listed as variables: first-listed diagnosis, all-listed diagnoses, days of care, and procedure. The second difference is that the demographic characteristics or groups (e.g., male, female, race) are listed as variables before 1988 but as values of variables afterward. The third difference is that the RSE parameters are expressed as percentages from 1979 through 1987 but as function coefficients afterward. The percentage format before 1988 is unsuitable for calculating CIs on some statistical software (e.g., SUDAAN [Research Triangle Institute, Research Triangle Park, NC]) (MF Owings, NCHS, written communication, May 2003). The fourth difference is that the listed RSEs before 1988 correspond only to a few specific weighted estimates, so interpolation is necessary to compute SEs and CIs for weighted sample estimates between these listed estimates. During or after 1988, however, the RSE parameters are function coefficients that allow direct computation of SEs and CIs of any weighted sample estimate. The parameters in either type of RSE table can be applied for SE and CI computations for data reports only if the unweighted number of hospital discharges is 30 or more; otherwise, the data would not be reliable (4).

Given these difficulties, manual computations will be inefficient and prone to error, particularly for studying multiple diseases over many years. For example, calculating CIs for the annual totals of five diseases in five sociodemographic categories over 5 years would require 125 computations. The computations for these CIs are based on 125 RSE values calculated from 50 different parameters selected from RSE parameter tables. Therefore, we developed a SAS (SAS Institute Inc, Cary, NC) program that both retrieves appropriate RSE parameters corresponding to given weighted estimates and calculates SEs and CIs for annual totals, multiple-year summaries, and average annual totals of multiple years of NHDS data (excluding those for newborns). This paper describes the structure and functions of the program and presents the rationale for robust calculations of CIs.

## Methods

**Figure 1:**
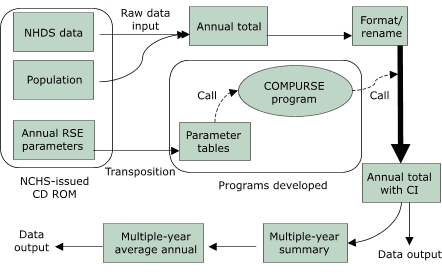
The process by which relative standard errors (RSEs), standard errors (SEs), and confidence intervals (CIs) are calculated for National Hospital Discharge Survey (NHDS) data (excluding those for newborns) using the COMPURSE program. NCHS indicates National Center for Health Statistics.

A flow chart shows how the SAS program performs RSE retrieval and SE and CI computation (Figure 1). The first part of the program includes a sampled hospital table from the NHDS instructions and transposes the 22 annual RSE parameter tables on the CD-ROM (4) into two new parameter tables by SAS array programming. The second part, the COMPURSE program, systematically retrieves the parameters corresponding to specified weighted estimates and automatically computes the required RSEs, SEs, and CIs. The last part of the program includes an interface for the user to provide information that allows the first two parts of the program to work. 

### Transposition of the parameter tables

Each NHDS annual sample is unique, and the values of the annual parameters differ by year, type of statistic, and group within each demographic category. Because the parameter formats before 1988 differ from those during and after 1988, the parameter tables for each time period were transposed separately. The 22 tables of RSE parameters (one for each year from 1979 to 2000) have been transposed with SAS array programming into two new parameter tables so that the new tables have similar overall structures. This allows the COMPURSE program to search systematically for parameters corresponding to the characteristics of the disease or condition of interest.

Before transformation, each parameter table of RSEs before 1988 contains separate subtables for the following three types of statistic: the first-listed diagnosis or all-listed diagnoses, days of care, and procedure. The parameters before 1988 are expressed as percentages of the point estimate that represent the RSE at two specific weighted estimates — the minimum and the maximum of a tabulated range. The weighted estimates are listed for the type of statistic: estimates from 5000 to 40 million for the diagnosis subtable, estimates from 10,000 to 250 million for the days-of-care subtable, and estimates from 5000 to 30 million for the procedure subtable. After transformation, within each type of statistic, RSEs are specified for characteristics or group (e.g., white, black, Asian/Pacific Islanders, type of hospital) within categories such as race, hospital, and geographic region. An "ALLOTHER" category identifies RSEs appropriate for all other hospital and patient characteristics (Table 2). Therefore, the transposed annual parameter tables are indexed by four variables: "YEAR" (year of the NHDS survey), "OUTCOME" (the type of statistic), "CATE" (demographic or geographic category), and "CHARACTE" (specific demographic characteristics or groups within each demographic category). For example, in the 1979 NHDS, the RSE for the number of hospital discharges with a specific diagnosis (i.e., a given International Classification of Diseases, Ninth Revision [ICD-9], code[s]) found among the type of statistic of all-listed diagnoses [ADX] for blacks would be 17.3% for the lowest weighted estimate of 5000 and 14.3% for the highest weighted estimate of 10,000 and indexed under [YEAR] = 1979, [OUTCOME] = ADX, [CATE] = RACE, and [CHARACTE] = BLACK (Table 2).

During or after 1988, the tabled parameters are not presented as percentages but as two coefficients of a function (5) to calculate RSEs for any weighted estimate size where the unweighted number of hospital discharges is 30 or more. Each of the four types of statistic (first-listed diagnosis, all-listed diagnoses, days of care, and procedure) has its own pair of coefficients. More patient characteristics and categories (e.g., regions, sources of payment, age, sex, race) are listed during or after 1988 than were listed before; "TOTAL" is listed for overall totals and all other nonspecified categories and characteristics (Table 2). For example, the RSE for the number of hospital discharges of a specific procedure for women in 1988 would be calculated from two coefficients, 0.00332 (a) and 467.482 (b), indexed as [YEAR] = 1988, [OUTCOME] = PC, [CATE] = SEX, and [CHARACTE] = FEMALE (Table 2).

Because the NHDS CD-ROM tables list annual RSEs expressed as percentages for given listed estimates for years 1979 through 1987 but list function coefficients for 1988 and afterward, the COMPURSE program must compute the RSE, SEs, and CIs differently for each period (5). Although the current program is limited to the years available on the CD-ROM (1979–2000), other transposed parameter tables can be added by extending the existing tables beyond 2000 (4). Because there are no error curves for NHDS data from 1965 to 1978, the COMPURSE program is unusable for data in these years. A third parameter table lists the annual number of hospitals sampled from 1965 to 2000 to compute CIs for the average annual totals of multiple-year summaries before 1988 (5). 

### Calculation of RSEs, SEs, and CIs

**Figure 2:**
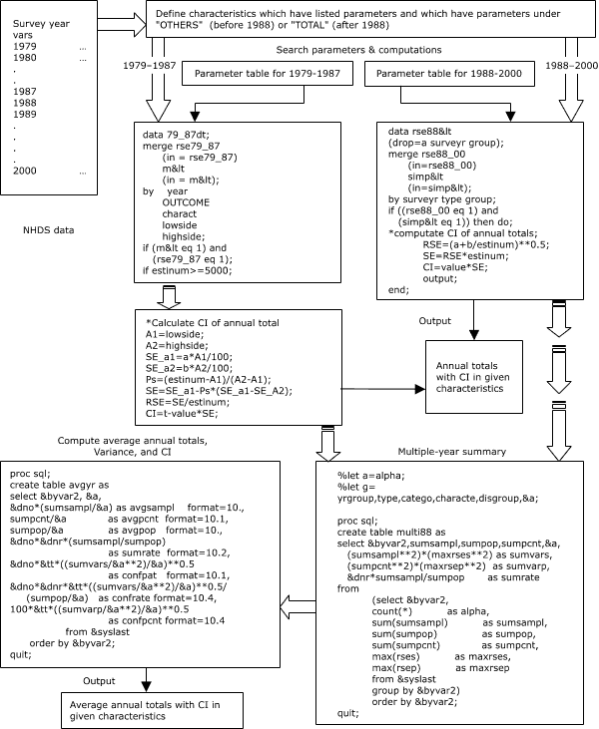
Main sections of COMPURSE program for selecting parameters and for computing relative standard errors (RSEs), standard errors (SEs), and confidence intervals (CIs) for annual totals and average annual totals of multiple-year summaries.

The second part of the COMPURSE program is a SAS program (version 6.12 or later) that searches for the appropriate parameter from the transformed tables and calculates the corresponding point estimates, SEs, and CIs by year, type of statistic, hospital and demographic category, and characteristics or groups (Figure 2). Depending on year, the program distinguishes the kind of RSE parameters (percentages or function coefficients) and the characteristics with specific parameters from those without specific parameters (e.g., "ALLOTHER" before 1988 or "TOTAL" during or after 1988).

COMPURSE merges user-specified data and the corresponding RSE parameter tables to look up specific values in the parameter tables. If survey year, type of statistic, category, and characteristics (group) within a category for the user-specified data agree with those from the corresponding RSE parameter tables, the program then selects the corresponding pair of parameters from the parameter tables. Before 1988, the COMPURSE program linearly interpolates between the RSE percentage values corresponding to the listed estimates above and below the weighted estimate (ESTINUM) the user specifies (5). During or after 1988, COMPURSE immediately calculates these intervals from the function coefficients selected during the table lookup for the weighted estimate (ESTINUM) the user specifies (5).

For annual totals with specified characteristics, COMPURSE can output the number, rate, and percentage of hospital discharges with their corresponding SEs and CIs (Appendix A). COMPURSE also provides another option to compute average annual totals for multiple years and their SEs and CIs (based on the third set of transposed parameter tables for years before 1988 or the function coefficients for 1988 and thereafter). The methods for computing these latter multiple-year averages are described in the NCHS documentation for the NHDS 1979–2000 data (5).

### User interface

**Figure 3:**
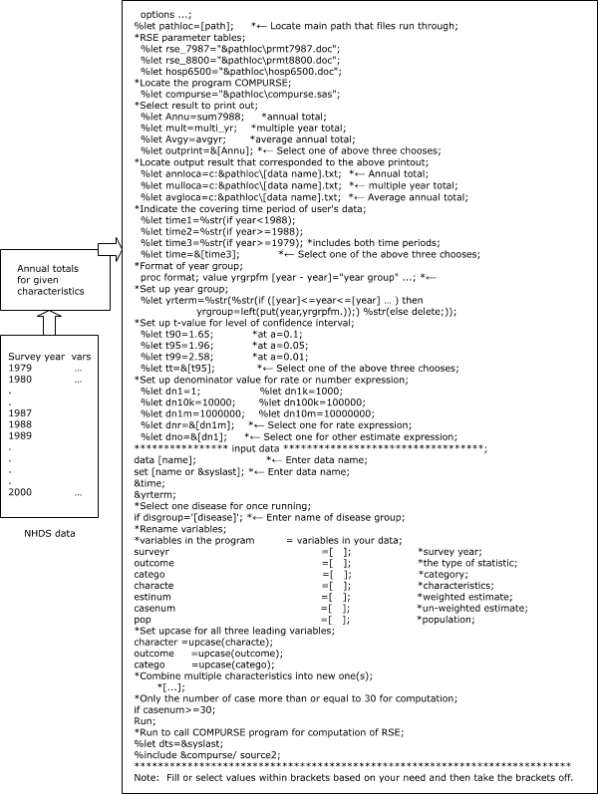
Main components of the user interface program for COMPURSE.

The third part of the program, the user interface, allows the user to define the time period for multiple-year summaries, to supply the normal deviate corresponding to the significance level for the CIs, to choose units for expressing the rate and the number of hospital discharges, and to provide other parts of the program with the location of files and the type of data input and output (Figure 3; Appendix B).

## Results

We tested the COMPURSE program with three data sets extracted from the NHDS, one from a publication (6) and the other two from projects on which the first author is working. The COMPURSE program was designed to perform a statistical analysis once for each disease or disease group. Analyzing multiple diseases in one program run requires the addition of SAS macro statements to the interface program. With the NHDS data cited (6), we tested the first 25 of 50 diseases in the year 2000. The estimates and their SEs computed with the COMPURSE program overall and by four age groups are compatible with those published by Hall and Owings (6) using SUDAAN software (Table 3). The COMPURSE results for annual and multiple-year summaries from 1988 to 2000 for arthritis and multiple-year summaries from 1979 to 2000 for epilepsy or seizure disorders are also compatible with manual computations (data not shown).

## Discussion

When reporting results from this program, the user should consider NCHS guidelines for reporting NHDS estimates. Because of the complex sample design of the NHDS, the NCHS recommends the following: 1) if an estimate is based on 29 or fewer unweighted sampled discharges, the value of the estimate should not be reported; 2) if this number is from 30 through 59, the value of the estimate may be reported but should not be considered reliable; 3) if this number is 60 or more, and if the RSE is less than 30%, the value of the estimate is reliable and may be reported; and 4) if the RSE of any estimate exceeds 30%, no matter what the number in the unweighted sample is, this estimate is unreliable and should not be reported. The NCHS further indicates that the user of the data should decide whether or not to report an estimate. However, if the user chooses to report an unreliable estimate, the user must inform the consumer (for example, a reader or a policy maker) that the estimate is unreliable (5).

If the overall number of hospital discharges for a disease of interest is small, the RSE may be relatively large. To reduce such large RSEs, the data analyst can aggregate multiple years of data to increase the number in the unweighted sample. However, such aggregation may defeat the purpose of the analysis (e.g., looking for time trends). 

Finally, computations of RSEs, SEs, and CIs cannot be applied to subgroups that combine different demographic groups (e.g., white males, black females). Computations can only be applied to single-category groups such as only whites or only males (MF Owings, NCHS, written communication, May 2003).

COMPURSE was programmed based on the National Hospital Discharge Survey 1979–2000 Multi-Year Public-Use Data File Documentation (5). However, it can also be used for data after 2000 as long as the RSE parameter table for these years is transposed and added to the new RSE parameter tables. Because there are no error curves for NHDS data from 1965 to 1978, the COMPURSE program is unusable for data in these years. The 1979–2000 transposed parameter tables, the COMPURSE program, and the data interface program described in this article are available from the first author, who will update and transpose parameter tables issued by NCHS for years after 2000. The program will be updated and modified to account for any discrepancies found in the future. Users who identify problems with the program or incorrect results should contact the first author.

## Figures and Tables

**Table 1:** Differences in Layout of Relative Standard Error (RSE) Parameter Tables Before 1988 and During or After 1988, CD-ROM on National Hospital Discharge Survey Data, 1979–2000^a^^,^ b

**1979–1987**				

**Type of Statistic for Hospitalizations (Listed as Subtables)**	**Weighted Estimates**	**Demographic Characteristics (Listed as Variables)**		

		**Region**	**Race**	**All Others**
First- or all-listed diagnosis	5,000–40,000,000	*	*	*
Days of care	10,000–250,000,000	*	*	*
Procedure	5,000–30,000,000	*	*	*

**1988–2000**								

**Demographic Characteristics (Listed as Values of Variables)**	**Type of Statistic for Hospitalizations (Listed as Variables)**							

	**First-listed Diagnosis**		**All-listed Diagnoses**		**Days of Care**		**Procedure**	

	**A**	**B**	**A**	**B**	**A**	**B**	**A**	**B**
Region	•	•	•	•	•	•	•	•
Race	•	•	•	•	•	•	•	•
Male	•	•	•	•	•	•	•	•
Female	•	•	•	•	•	•	•	•
Aged <15 years	•	•	•	•	•	•	•	•
Other	•	•	•	•	•	•	•	•
**TOTAL**	•	•	•	•	•	•	•	•

a CD-ROM issued by the National Center for Health Statistics, 2002. Relative standard error (RSE) parameters are expressed as percentages before 1988; asterisks (*) represent possible RSE values during 1979–1987. In contrast, each statistic for 1988–2000 has two coefficients, A and B, which are derived from RSE curves.

b Bullets (•) represent coefficient values during 1988–2000.

**Table 2:** Examples of Relative Standard Error (RSE) Parameter Tables Transformed by COMPURSE Program Using Data From National Hospital Discharge Survey, 1979–2000^a^

**1979–1987**							

**Sample Year**	**Type of Statistic^a^**	**Demographic or Geographic Category**	**Description of Category**	**Characteristics**	**Description of Characteristics**	**Parameters^b^**	

**[VARIABLE]=[YEAR]**	**[VARIABLE]=[OUTCOME]**	**[VARIABLE]=[CATE]**		**[VARIABLE]=[CHARACTE]**		**A-RSE (%)**	**B-RSE (%)**
1979	ADX	ALLOTHER	All others	ALLOTHER	All others	17.3	14.3
1979	ADX	BED_NUMB	Number of beds	BEDLS100	Beds below 100	23.1	19.2
1979	ADX	HOSPITAL	Hospital ownership	GOVERNMT	Government	28.7	24
1979	ADX	HOSPITAL	Hospital ownership	NONPROFT	Nonprofit	15.8	13.9
1979	ADX	HOSPITAL	Hospital ownership	PROPRIET	Private	28.7	24
1979	ADX	RACE	Race	AMER_IND	American Indian	No data^c^	No data^c^
1979	ADX	RACE	Race	ASIA_PAC	Asian/Pacific Islander	No data^c^	No data^c^
1979	ADX	RACE	Race	BLACK	Black	17.3	14.3
1979	ADX	RACE	Race	MULT_RAC	Multiple races	No data^c^	No data^c^
1979	ADX	RACE	Race	NOTSTATE	Not stated	25.1	22.9
1979	ADX	RACE	Race	OTHERS	Others	No data^c^	No data^c^
1979	ADX	RACE	Race	WHITE	White	17.3	14.3
1979	ADX	REGION	Region	REGION	Region	25.3	21.2

**1988–2000**							

**Sample Year**	**Type of Statistic^a^**	**Demographic or Geographic Category**	**Description of Category**	**Characteristics**	**Description of Characteristics**	**Parameters^b^**	

**[VARIABLE]=[YEAR]**	**[VARIABLE]=[OUTCOME]**	**[VARIABLE]=[CATE]**		**[VARIABLE]=[CHARACTE]**		**A-RSE (%)**	**B-RSE (%)**
1988	PC	AGE	Age	15-44	Aged 15-44 y	0.00362	443.165
1988	PC	AGE	Age	45-64	Aged 45-64 y	0.00374	463.928
1988	PC	AGE	Age	65-UP	Aged ≥65 y	0.00351	442.05
1988	PC	PAYMENT	Source of payment	MEDICAID	Medicaid	0.00962	365.296
1988	PC	PAYMENT	Source of payment	MEDICARE	Medicare	0.00435	421.248
1988	PC	PAYMENT	Source of payment	NOCHARGE	No charge	0.02929	312.749
1988	PC	PAYMENT	Source of payment	NOTSTATE	Not stated	0.06001	345.075
1988	PC	PAYMENT	Source of payment	OTHERGOV	Other government	0.04491	343.602
1988	PC	PAYMENT	Source of payment	PRIVATE	Private	0.0035	405.275
1988	PC	PAYMENT	Source of payment	SELFPAY	Self-pay	0.01461	249.645
1988	PC	PAYMENT	Source of payment	BCBS	Blue Cross/Blue Shield	No data^c^	No data^c^
1988	PC	PAYMENT	Source of payment	HMO/PPO	HMO/PPO	No data^c^	No data^c^
1988	PC	PAYMENT	Source of payment	WORKCOMP	Worker’s company	0.03702	509.025
1988	PC	RACE	Race	ALLOTHER	All others	0.00842	361.469
1988	PC	RACE	Race	BLACK	Black	No data^c^	No data^c^
1988	PC	RACE	Race	NOTSTATE	Not stated	0.04382	522.318
1988	PC	RACE	Race	WHITE	White	0.0038	477.624
1988	PC	REGION	Region	MIDWEST	Midwest	0.01138	464.393
1988	PC	REGION	Region	NORTHEAS	Northeast	0.00493	285.834
1988	PC	REGION	Region	SOUTH	South	0.00833	449.5
1988	PC	REGION	Region	WEST	West	0.01193	571.693
1988	PC	SEX	Sex	FEMALE	Female	0.00332	467.482
1988	PC	SEX	Sex	MALE	Male	0.00376	428.402
1988	PC	TOTAL	All others or total	TOTAL	All others or total	0.00415	464.814

a ADX indicates all-listed diagnoses; PC, procedure. Alternatives for type of statistic: DC indicates days of care; FDX, first-listed diagnosis.

b Before 1988, parameter A represents the RSE value corresponding to the lowest weighted estimate of 5000 (the limit of an interval possibly containing the actual weighted estimate), and parameter B represents the RSE value corresponding to the second lowest weighted estimate of 10,000 (the limit of another interval possibly containing the actual weighted estimate). Linear interpolation between these RSE values is necessary to estimate RSE values for weighted estimates between these tabulated estimates. However, during or after 1988, the parameters A and B represent individual coefficients of a function.

c Values missing in National Hospital Discharge Survey.

**Table 3:** Calculations Using COMPURSE Compared With Calculations Using SUDAAN^a^ ^b^

**Category of First-Listed Diagnosis**	**All Ages**			**Aged 0-14 y**			**Aged 15-44 y**			**Aged 45-64 y**			**Aged ≥65** **y**		

	**N**	**SE**	**SER**	**N**	**SE**	**SER**	**N**	**SE**	**SER**	**N**	**SE**	**SER**	**N**	**SE**	**SER**
All conditions	31,706	1,520	1,218	2,383	349	328	9,969	482	405	6,958	351	290	12,396	713	555
Infectious and parasitic diseases	787	40	42	160	24	26	173	11	12	150	10	10	305	20	18
Septicemia	326	18	20	16	3	3	32	3	5	62	5	6	216	15	14
Neoplasms	1,587	79	70	37	6	11	289	16	14	566	31	26	695	42	38
Malignant neoplasms	1,156	58	54	27	5	8	120	8	8	393	22	19	617	38	33

**Category of First-Listed Diagnosis**	**All Ages**			**Aged 0-14 y**			**Aged 15-44 y**			**Aged 45-64 y**			**Aged ≥65**		

	**R**	**SE**	**SER**	**R**	**SE**	**SER**	**R**	**SE**	**SER**	**R**	**SE**	**SER**	**R**	**SE**	**SER**
All conditions	1140.1	54.7	43.8	393.9	57.7	54.2	815.9	39.5	33.2	1141.7	57.6	47.6	3595.5	206.8	161.1
Infectious and parasitic diseases	28.3	1.5	1.5	26.4	4.0	4.4	14.2	0.9	1.0	24.6	1.6	1.7	88.5	5.9	5.2
Septicemia	11.7	0.7	0.7	2.6	0.5	0.5	2.6	0.3	0.4	10.2	0.9	1.0	62.7	4.4	4.2
Neoplasms	57.1	2.8	2.5	6.1	1.0	1.8	23.7	1.4	1.1	92.9	5.1	4.3	201.6	12.4	10.9
Malignant neoplasms	41.6	2.1	1.9	4.5	0.8	1.3	9.8	0.7	0.6	64.5	3.7	3.2	179.0	11.1	9.6

a The number (N) and rate (R) of discharges from short-stay hospitals by first-listed diagnosis and age, United States, 2000. N is expressed per 1000. R is expressed per 10,000.

b The number (N) of discharges and standard errors in reference (SER) of five diseases were cited from Hall and Owings (6). SER was calculated with SUDAAN. N, R, and standard error (SE) were calculated with COMPURSE.

## Appendices

### Appendix A. Calculating Annual Totals for a User-specified Weighted Estimate

The procedure and equations to calculate RSE, SE, and CI of annual total for a weighted estimate (ESTINUM) specified by the user are as follows:

For those before 1988:
A1=lowside;
A2=highside;
SE_a1=a*A1/100;
SE_a2=b*A2/100;
Ps = (ESTNUM - A1)/(A2 - A1);
SE = SE_a1 - Ps*(SE_a1 - SE_a2);
RSE = SE/ ESTNUM;
CI = t-value*SE;

where A1 and A2 represent the listed estimates in the RSE table which are at the low side and high side most adjacent to the weighted estimate (ESTINUM); a and b are RSE values corresponding to the two listed estimates; Ps is a ratio of the difference between the weighted estimate and the listed estimate in low side over that between listed estimates in the high side and the low side; SE_a1 and SE_a2 are SE for the listed estimates in the low side and high side, respectively, and SE is the standard error of the weighted estimate the user specified; RSE is relative standard error and CI is confidence interval; t-value is the t value at the given statistical level.

For those during or after 1988:
RSE = (a+b/ESTINUM)0.5;
SE = RSE*ESTINUM;
CI = t-value*SE;

where a and b are coefficients listed in the RSE parameter tables, and the other components are the same as those in the previous equation.

### Appendix B. Program Instruction

For annual totals with specified given characteristics, COMPURSE can output the number, rate, and percentage of hospital discharges with their corresponding SEs and CIs. COMPURSE also provides another option to compute average annual totals for multiple years and their SEs and CIs (based on the third set of transposed parameter tables for years before 1988 or the function coefficients for 1988 and thereafter). The methods for computing these latter multiple-year averages are described in the NCHS documentation for the NHDS 1979–2000 data (5).

The COMPURSE package includes three parts: 1) three sets of transposed parameter tables and the example reference table of variables (Table 2) for data preparation; 2) the COMPURSE program for parameter retrieval and confidence interval calculation (Figure 2); and 3) the interface program (Figure 3), which the user modifies to provide information to run the other two parts of the package.

The user should first copy all three parts of the COMPURSE package to the user’s computer. Specifically, the user should save without changes the transformed parameter tables and the COMPURSE program in a directory on the computer hard drive. The user should save a copy of the interface program under a new name for the user’s current analysis and leave the original copy to be copied for next analysis.

Second, the user should change this new copy of the interface program to define the computer directory path where the RSE parameter tables and the COMPURSE program for CI computations are located. In this new copy, the user should also change the statements in brackets, optionally typing in appropriate words, or the sentences ending in ellipses. For example, to set up yearly groups in value statement of PROC FORMAT, to modify for the new copy of the interface program, or to combine multiple values in variable characteristics into a new value (cf., Figure 3).

These changes are the following:

1. The name(s) of the path(s) to the hard disk directories where the three parameter tables are located;
2. The name of the hard disk directory path for the COMPURSE program;
3. The name(s) of the hard directory path(s), file name(s), and extension(s) for the location(s) to print the output results for either the annual totals, the average annual totals of multiple year summaries, or both. If the user wants only one of the latter sets of results, the user should comment out the location of the other set of results by typing an asterisk at the start of the corresponding line.

Third, the user must specify which of three time periods should be analyzed: 1979–1987, 1988–2000, or 1979–2000. For multiple-year summaries, the time period specified should be the same for the hospitals sampled and for the data selected. The COMPURSE program will compute SEs and CIs for average annual totals for such summaries even if the time period spans the transition period 1987–1988.

Fourth, the user can specify a confidence level for the Cl different from the default (95%) by typing an ampersand (&) and either of two other options, t90 (90% level) or t99 (99% level), after the macro variable, &tt. The user can also specify different options for saving the output results of hospital discharges and for changing the magnitude of rates by selecting denominators for the rate (DNR&[any of the listed names of the numbers]) or for the number (DNO&[any of the listed names of the numbers] or for both.

Fifth, the user should input the extracted data, with the following restriction: the COMPURSE program processes the relevant estimates, their SEs, and their CIs only for one disease or disease group at a time; however, the user may write a macro in the user interface program to compute SEs and CIs for more than one disease or disease group at a time.

The user’s extracted data should include both diseases and years of interest from the NHDS CD-ROM (1979–2000) and the annual weighted sample estimates of hospital discharges by specified characteristics in separate external files accessible by SAS. These data may be input through SAS and include two more variables. The first step is to determine what type of statistic the user is interested in — first-listed diagnosis, all-listed diagnoses, procedure, or days of care. The second is to define the category for the characteristics. The RSE parameter table (Table 2) will be helpful for selecting these types of statistic and characteristics. For example, if the user were interested in the first-listed diagnosis of hospitalizations among those aged >15 years, the value of the first variable for the type of statistic (OUTCOME) should be “FDX” (first-listed diagnosis), and the values of the second variable (characteristic for the type of statistic) for the category variable (CATE) should be “AGE.”

Although the user can name variables in the input file in the desired way, the program uses a standard set of variable names. The user should type in the data variable names from the right side of the assignment statements for the type of statistic and their characteristics (the words in the brackets of the interface program, cf., Figure 3). The program uses the standard variable names on the left side of these assignment statements. For example, if the variable name representing the survey year in the user’s data is “YR,” the user should fill in the bracket on the right side of the assignment statement with YR. If the variable name representing the weighted estimate of hospitalizations in the user’s data is “weitnum,” the user should fill in the bracket on the right side of the assignment statement with WEITNUM. If the user does not want to compute a rate, the user can fill in the bracket with a period (SAS’s missing value indicator). The unweighted estimate (unweighted number of discharges) is named as “CASENUM” in the program. The user should calculate this number and include it on the input file. The computations of CIs are conducted only if the unweighted estimate is ≥30. If some characteristics have too few hospital discharges to be observed individually, they can be summarized as a new value under the variable name CHARACTE (characteristics). The new character value should not have the same name as any of the other values in the assignment statements mentioned previously. Finally, the user should remove all the brackets from the modified copy of the user interface program before running the program.

After specifying the NCHS data set of interest and any of the previous options, the user can submit the copy of the user interface program through SAS. This interface program in turn calls the COMPURSE program through the %INCLUDE &COMPURSE statement to calculate the estimates and their RSEs, SEs, and CIs.
